# Supplementary material for: The Permeability and Selectivity of the Polyamide Reverse Osmosis Membrane were Significantly Enhanced by PhSiCl3
Source: Membranes (Basel). 2021 Feb 18;11(2):142. doi: 10.3390/membranes11020142 (PMC7922360; doi:10.3390/membranes11020142)
Supplement: Supplementary file 1 [file membranes-11-00142-s001.pdf]

membranes

MDPI

Article

Supplementary Information: The Permeability and Selectivity of the Polyamide Reverse Osmosis Membrane were Significantly Enhanced by $PhSiCl_3$

Junjie Yu, Kaifeng Gu, Binbin Yang, Kaizhen Wang, Yong Zhou * and Congjie Gao

1. TEM Images of TFN and TFC Membranes

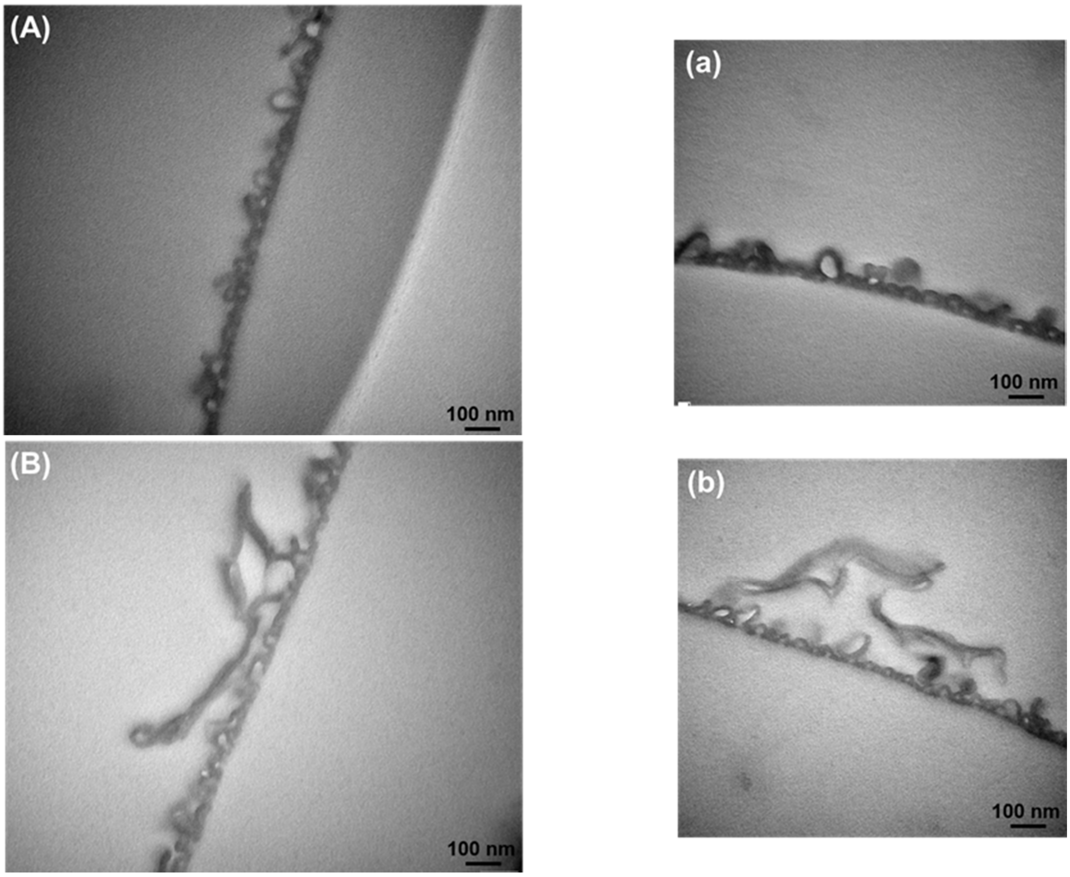

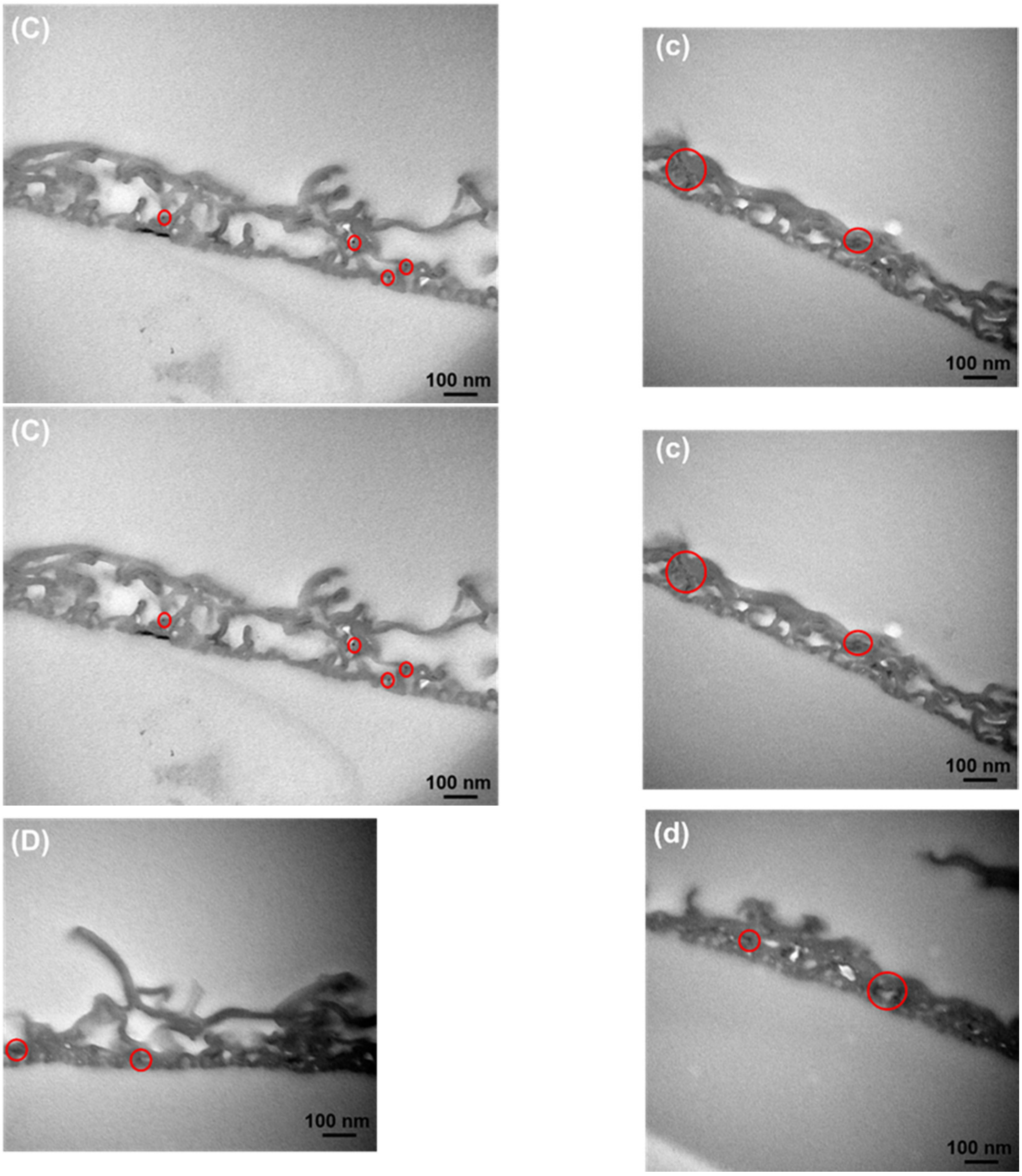
(C)
100 nm
(c)
100 nm
(C)
100 nm
(c)
100 nm
(D)
100 nm
(d)
100 nm

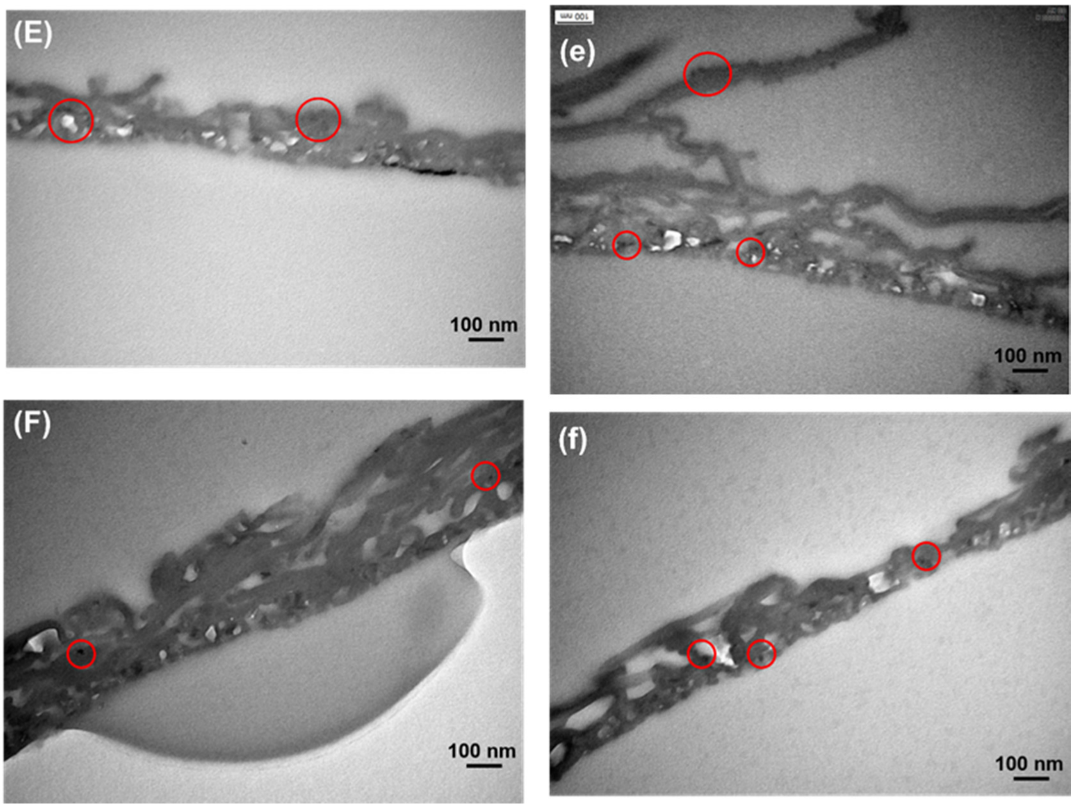

Figure S1. TEM images of the membranes with different PTS loading: (**A**, **a**) TFC membrane; (**B**, **b**) TFN-0.1 membrane; (**C**, **c**) TFN-0.2 membrane; (**D**, **d**) TFN-0.3 membrane; (**E**, **e**) TFN-0.4 membrane; (**F**, **f**) TFN-0.5 membrane.

We can see that with the increase of the concentration of PTS, the protuberance of TFN membrane is higher and higher, and the cross-section structure is more and more clear. This can be attributed to the introduction of silicon element and cavity, which increases the contrast of cross-section TEM image.

2. PA Layer Transfer Method

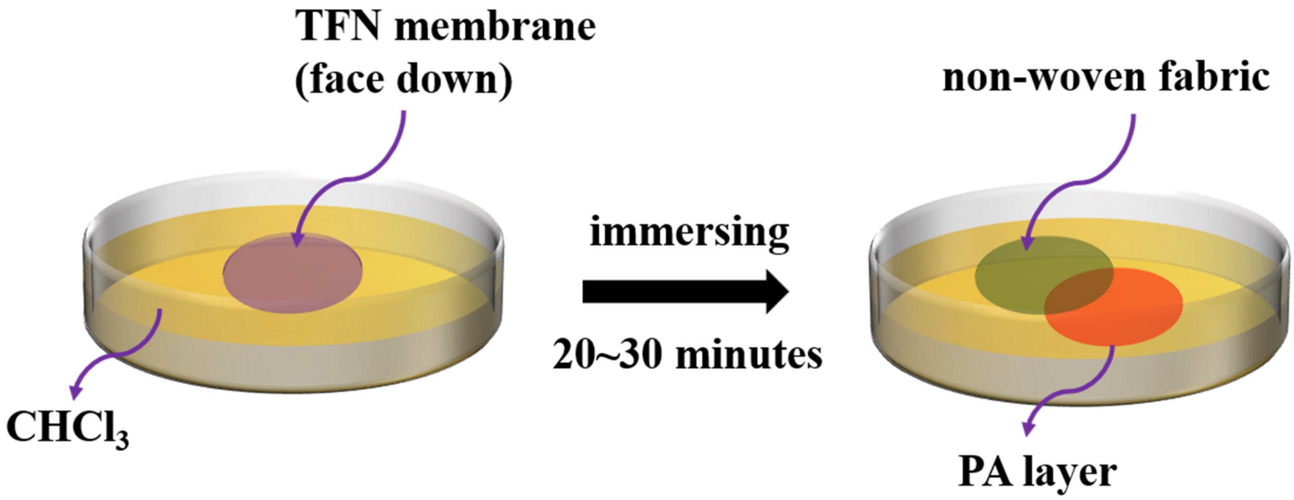

Figure S2. PA layer back transfer process.

Chloroform ($CHCl_3$) is used to dissolve the PSF base layer (Figure S2). Place the TFN membrane face down and float it in chloroform. After the PSF is dissolved, the non-woven fabric is separated from the PA layer. Then, we deposit the floating PA layer on a silicon wafer. The membrane surface structure on the silicon wafer is the PA back.

3. SEM Image of PSF Membrane

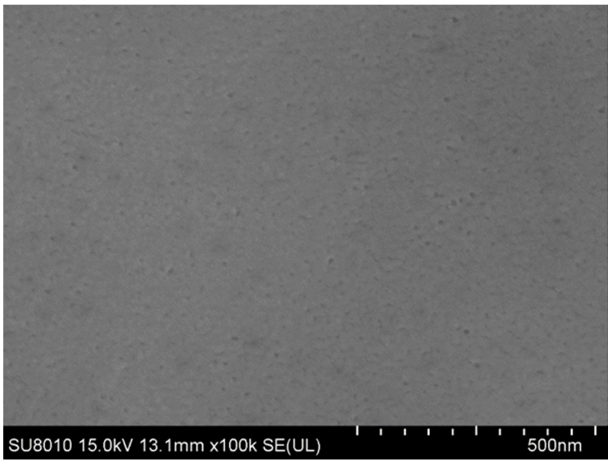

Figure S3. SEM image of PSF membrane.

The PSF base membrane has a smooth surface without special structure (Figure S3). In comparison, TFN membrane has a special fold structure (Figure 1B–F), which forms a unique transfer process.

4. Aperture Fitting

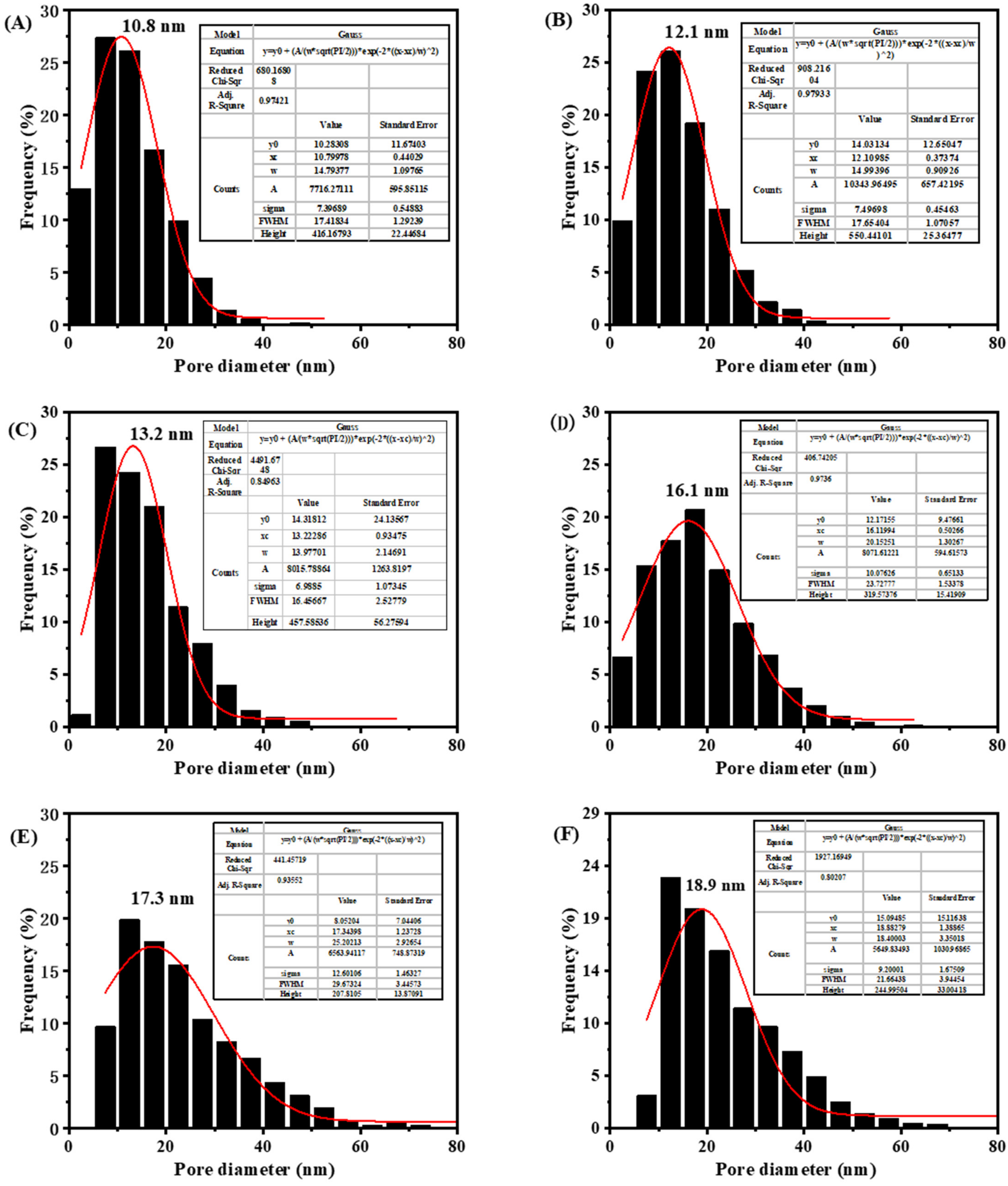

Figure S4. Pore size distribution fitting curve.
